# A Treatise on the Epidemic Cholera; Containing Its History, Symptoms, Autopsy, Etiology, Causes, and Treatment

**Published:** 1833-05

**Authors:** Alexander Turnbull Christie

**Affiliations:** the Honourable East-India Company's Service; Member of the Royal Asiatic Society of Great Britain and Ireland, of the Wernerian and Royal Medical Societies of Edinburgh, &c.


THE LONDON

### Medical and Physical Journal.

411, VOL. LXIX.] MAY 1833. [83, *New Series*.

"For many fortunate discoveries in medicine, and for the detection of numerous errors, the world is indebted to the rapid circulation of Monthly Journals; and there never existed any work to which the Faculty in Europe and America, were under deeper obligations than to the 'Medical and Physical Journal of London,' now forming a long but an invaluable series."—RUSH.

### ORIGINAL PAPERS.

### THE EPIDEMIC CHOLERA.

*A Treatise on the Epidemic Cholera; containing its History, Symptoms, Autopsy, Etiology, Causes, and Treatment.* By ALEXANDER TURNBULL CHRISTIE, M.D., of the Honourable East-India Company's Service; Member of the Royal Asiatic Society of Great Britain and Ireland, of the Wernerian and Royal Medical Societies of Edinburgh, &c.

(Continued from page [illegible]) 283.)

WE have already observed that the epidemic reached the most southerly point of the Peninsula of India in January 1819; and it was at this time it first appeared in the Island of Ceylon, where Colomba was the first town attacked by it. As usual, it gradually spread in all directions, broke out in Trincomalee in April, and in Point de Galle in July, and had visited almost every part of the island before the end of the year.

It has been said that the town of Trincomalee was infected with the disease by the crew of the ship Leander, in July 1820, who were suffering from it before their arrival, and had caught it at Pondicherry; but there is little reason for supposing this to have been the case, the disease having already existed in the island for more than a year, and Trincomalee having been once before visited by it. It has been also supposed to have been introduced by means of contagion into the island of Mauritius, where it first made its appearance in September 1819; but it is sufficient to state, in opposition to this notion, that the medical commission appointed by the government of that settlement, to investigate the nature of the disease, gave it as their opinion that it was not contagious. It found its way to the island of Bourbon in January 1820. The government had taken all the precautions in their power to prevent its introduction into the island, and denounced the penalty of death against any person found clandestinely landing on its shores; but all to no purpose. It must be remarked, however, that it has been supposed that the disease had been introduced by means of a communication having taken place between a boat from the shore and a vessel named the Picvar, which arrived on the 7th of January from Port Louis, in the Mauritius, where the disease then prevailed.

We have now arrived at that part of our narrative which demands our most anxious attention, viz. which exhibits the disease spreading from India to the west, and thus approaching nearer and nearer to our own homes. In the summer of 1821, it raged with tremendous violence along both sides of the Persian Gulph, especially at Muscat, Bushire, and Bussorah, and at the latter place is said to have carried off 14,000 people in fifteen days. It reached Shirauz in September, and numbered among its victims the mother, one of the wives, and a child, of the Prince of Persia, besides many Georgian females; and 6,000 deaths were counted out of a population of 40,000. It reached Bagdad about the same time; it then subsided considerably during the winter-months, but burst forth with fresh fury in the summer of 1822, when it attacked Teheran, and spread all over Azorbijan. It was at this time that the Prince Abbas Mirza was carrying on the war against the Turks on the high table-land near the source of the Euphrates, when both armies were destined to suffer more severely from an invisible scourge than from the swords of their antagonists. The following account of the occurrence of the epidemic in the Persian army, after the battle of Toprah Kullah, is extracted from the travels of Mr. Baillie Fraser. "The Prince pursued his success as far as the pass of Deear, about three days' march from Topra Kullah, when the epidemic cholera, which had appeared in his camp even previous to the action, now broke out violently, and he thought fit to commence a retrograde movement to Khoace. From that moment the Persian army also appears to have been virtually dissolved; the men dropt off rapidly, and whole troops deserted to return to their homes, so that, by the time he reached Kooe, he had scarcely any army to dismiss. The loss by disease during this retreat was certainly great, but has been variously represented. The most probable accounts set it down at about four thousand men, or about a tenth part of the whole force; in some battalions three hundred out of one thousand died, and the rear of their line of march was strewed with dead bodies, as if it had been all the way in action." (P. 315.)

"It is difficult to say how or from whence the epidemic cholera, that scourge of the East, reached Tabreez. It was supposed to have reached from Bagdad along the caravan-road, by Hamadan and Sennar, but no accounts to be at all depended on could be obtained of its gradual progress. A whisper had gone forth that the disease had appeared in the town as early as the 12th of July; but a week made it no longer doubtful, and upon our return to Tabreez on the 24th, after a short absence, we understood that from fifteen to thirty were daily dying of cholera.

"A panic now commenced, and the authorities of the place, instead of endeavouring to restore confidence, and maintain order in the prince's absence, encouraged the inhabitants to quit the city. The old Caimoakan set the example, by ordering away the prince's harem, and announcing his intention to follow it; he did not, however, in his alarm, neglect his English friends, but sent a message advising them to follow his example, and offering a garden in the country for their accommodation.

"During the next ten days the state of the distemper varied greatly; sometimes the fatal appearances diminished so much, that it was even doubted whether the alarm had not been a false one; the sick were attacked with vertigo and sickness, which was attributed to the powerful effect of the sun's rays; and, though some died, yet more recovered, without having evinced many of those more peculiar and alarming symptoms which generally mark the disease. By degrees, however, these symptoms disappeared; violent vomiting, accompanied with painful cramps, damp clammy sweat, cold and bloodless extremities, burning heat at stomach, a sunken death-like countenance, cessation of all the pulses: these, and other striking characteristics of this fearful disease, marked the fatal cases, and terminated in death.

"Often when the disease was at its height, the first seizure, indicated by vertigo, proved fatal at once. Several instances came to our knowledge of persons thus attacked in the streets, who fell down senseless, and never recovered.

"The treatment pursued by the natives was, in all cases, the same which we had remarked in other parts of Persia. The moment a person was attacked, they seized him, and drenched him in cold water, made colder by ice, which is abundant in Tabreez; verjuice was thrust down his throat when the unhappy patient could swallow, and ice was often heaped upon the stomach. A chill was thus given which probably aided the disease, the nature of which is to drive the circulation from the surface and extremities. This discipline, however injurious in cases of true cholera, was salutary and successful in cases of mere vertigo, which, from the heat of mid-day in the sun, was not unfrequent; and the occasional cures thus performed on persons supposed to be labouring under cholera, strengthened the faith of the natives in their own practice.

"During this time the natives, panic-struck, fled in crowds from their homes, until the city became a desert."*

It is remarkable, that about the same time that the epidemic broke out along the Persian Gulph, it also made its appearance in Khorassan, without in any instance being traceable to contagion; and is also said to have spread over great part of Arabia, to the holy cities of Mecca and Medina.† It even stretched as far as the eastern shores of the Mediterranean, and inflicted its scourge upon the towns of Aleppo, Antioch, Latichea, and Damascus.

The town of Astracan, which has since been so severely afflicted, received the epidemic for the first time in September 1823, when the Russian flotilla of the Volga also suffered. But it did not continue long, and was supposed to have been checked by the occurrence of a very intense frost. After the year 1823, the disease subsided in all the countries we have just mentioned, and for several years it had completely disappeared, excepting a few sporadic cases, which were always observed to be most prevalent during the autumn. Thus it continued till the year 1829, when it again invaded Teheran,‡ and other towns of Persia; and about the middle of the year it suddenly appeared in the town of Oremburg, situated on the river Oural, between the 51st and 52d north latitude, and at a time when all the surrounding countries for hundreds of miles were healthy, and had hitherto been exempt from the malady.

Again it received a check from the cold of winter; but it only lay dormant for some months, to revive with increased malignancy in the spring of 1830. It was in June that it appeared for the first time in Georgia. It had been raging in the Persian towns of Resht, Zingalak, and Touris, and about the middle of this month broke out in Saliany at the mouth of the river Kur, whence it spread into the provinces of Bakou and Koubac, into the Rhanat of Talyskh, to Derbent, into the province of Seleki, and the district of

* Pp. 316, 317. † Lieut.-Col. Monteith's MSS.

‡ M. Moreau de Jonnes is mistaken in supposing that this was the first invasion of the disease at Teheran; for it raged there in July 1822.—See "Letter from a Gentleman in Persia, &c." Asiatic Journal, 1823.

Elisabethpol. Up to the 21st of July, out of 4557 persons attacked in these different places, 2447 had been cured, 1655 had died, and 457 were still suffering. From the district of Elisabethpol the epidemic ascended the Kur, appeared in the environs of Tiflis on the 9th August, and between the 13th and 19th of that month it carried off 238 persons. It was supposed there not to be contagious, and that the best means to escape from it was to retire to elevated situations; and accordingly the authorities of Tiflis gave the inhabitants permission to seek a refuge in the mountains, and more than two-thirds of them left the city. The total number of cases at Tiflis from the 9th of August up to the 1st of October, when the disease had ceased, was 2222, of which 1575 had proved fatal, and the remainder had been cured.

From Saliany the epidemic took another direction, following the shores of the Caspian Sea and of the Volga. It broke out on the 17th July in the environs of Sedlistoff, and on the 1st August (o. s. 19th July) at Astrakan, when, in the course of ten days, out of 1229 persons who were attacked, 433 died; and afterwards the mortality had increased there to nearly a hundred a-day. It appeared on the 17th August at Tzeretzin, and on the 21st at Saratoff, and at the latter place raged with very great malignancy for nearly a month; for we find, by the official returns, that up to the 21st September it had carried off altogether 2367 individuals, of whom 1133 were men, 1011 women, 118 boys, and 105 girls.

It also extended its ravages into the province of the Caucasus, on both banks of the Terek, to Kizliar, and along the Kuma, to Novotcherkask, and to some places in the territory of the Don Cossacks, where it continued till the beginning of October; and it reappeared in the district of Oremburg, at the towns of Gourieff and Ouralsk.

At this time his Majesty the Emperor of Russia caused the most active measures to be adopted, to stop, if possible, the progress of the disease, and to afford relief to the sufferers. Numerous medical officers, with medicines, were sent into the provinces where the disease prevailed; a medical commission was appointed to investigate the nature of the epidemic, and to take whatever measures they might think fit for checking it; and Count Zakreusky, minister of the interior, was also sent into these provinces for a similar purpose.

Frequent accounts were now received at St. Petersburg of the progress of the disease, towards the north. It had penetrated about the beginning of September to Samara, on the Volga, in the government of Sumbirsk, and to Penza; and several boatmen had died of it on-board of the merchants'

boats at Nigini, Novgorod, towards the middle of September. It reached Kostroma and Yarosloff in the middle of this month, and carried off many victims in these and other towns of the same district. It also continued to spread through the Ukraine, and visited the towns of Kherkoff, Izume, &c. in the beginning of October.

The epidemic now approached close to the city of Moscow, and the military governor, Prince Galatzyne, hastened to take precautions against it. In order to prevent all communications with the inhabitants of the districts where the disease already existed, a military cordon was established along the frontiers of the government of Moscow: four passages only were left open, and there barriers of observation were stationed. Of the eighteen entrances to the city itself eight were closed, and all strangers who arrived at the others were directed to four stations, in order to undergo quarantine. Notwithstanding these careful measures, the disease made its appearance in the city, and the first cases are supposed to have occurred on the 28th September, two days before the above precautions had been taken. A commission was immediately appointed, consisting of two sections, the one administrative, the other medical, the former being charged with the duty of inspecting the different quarters of the town, and of taking measures for preventing the spread of the disease; the latter having to attend to its treatment, and to draw up and publish daily bulletins.

The city of Moscow contains 300,000 inhabitants, and it is remarkable that its sanitary condition was never more favorable than it was at this time, the average number of deaths amounting to only fifteen a-day, whereas they had generally averaged thirty.*

On the 11th of October, the governor-general of Moscow published the following letter, which he had received from his Majesty the Emperor.

"It is with the most profound grief I have received the news you have communicated to me. Make me acquainted by expresses, with the progress of the disease. My departure will be regulated by the communications you transmit to me. I will come to partake of your dangers and labours. Let us submit ourselves to the decrees of the Almighty! I approve of all the measures you have adopted. Thank, in my name, those who under these circum-

* From the month of September up to the present time, (January 1831,) official bulletins have been published in the Russian newspapers of the number of seizures, deaths, and recoveries, in the different governments; but such details would be inconsistent with the nature of the present work, which professes only to trace the general features and progress of the disease.

stances unite their efforts to yours. Meanwhile I build upon them my strongest hopes."

"*24th September* (O. S.), *or 6th October*."

The impression of this letter was scarcely complete when the Emperor himself arrived in Moscow, which produced the happiest effects upon the depressed spirits of the people. A still more vigorous quarantine was now enforced; and no one was permitted either to enter or depart from the city, except for the conveyance of provisions, for which certain market-places were exclusively appropriated. A double military cordon was also formed upon all the roads which lead to St. Petersburg, and placed under the command of experienced generals, to prevent, if possible, the communication of the disease to that capital.

Every precaution against infection which could be thought of was adopted; and, among other things, large quantities of the disinfecting substance, the chloruret of lime, were employed in all quarters, but to no purpose, the malady continuing its ravages. The inhabitants of Moscow displayed the greatest patriotism and philanthropy, in their endeavours to aid their suffering fellow-citizens. Large sums of money were subscribed, for the purchase of medicines and other necessaries; numerous hospitals were established, and many private houses were voluntarily given up to be converted into abodes for the sick.

In the beginning of November the disease began to decline in various parts of Russia, and had ceased in many of the towns of the southern provinces; but it again burst forth in the government of Astracan, among the Kalmuks of the Oulouss and Erksteneff, and in the horde of Kirghises, near Kven Peski.

On the 29th October, a committee of health, which had been established at Odessa, had its first meeting, to take into consideration the best means of preventing the introduction of the epidemic into that town. A few cases are said to have occurred between the 29th of this month and the 1st or 2d of November; the disease then subsided until the middle of November, about which time eleven cases were reported, seven of which proved fatal, and by the latest accounts* we learn that it has occasionally reappeared, and had not entirely ceased in the middle of December.

The disease having diminished considerably in Moscow in the beginning of December, the Emperor commanded the ex-

* Received in January 1831.

ternal cordon to be removed, all the internal arrangements, however, being still kept up.

From the first appearance of the disease in Moscow, between the 28th and 30th September, the number of cases gradually and slowly increased till the 20th or 22d October, from which time till the end of the month, the new cases averaged about two hundred a-day, and the deaths about half that number. This was the period of its greatest malignancy, and henceforward it began slowly but sensibly to diminish. During the first half of November, about ninety-five persons upon an average were attacked daily, of whom more than one-half, or nearly forty-five a-day, died. In the latter half of the same month, the number of attacks and deaths averaged respectively about forty-eight and twenty-two a-day. The disease had now very much abated, but still continued throughout the whole month of December, the average number of seizures amounting to fifteen, and of deaths to eight a-day. The total number of cases in Moscow, from the commencement of the epidemic, in the end of September, up to the 2d January 1831, was 6305, of which 3533 had proved fatal; a great mortality, when we consider that the sick had all the advantages of speedy medical aid, local hospitals, and all the resources of a great capital.

"Is the cholera which has spread over the greater part of Asia, and has been recently communicated to Russia, a new disease in Europe?" This is an important question, and must now demand our attention, being not merely one of speoulation, but one which is intimately connected with the theories which have been proposed concerning the nature and causes of the epidemic. It shall be shewn, in the following chapters, that there are two distinct kinds of cholera, to which I have applied the names of catarrhal and inflammatory (cholera catarrhalis* and cholera pyretica†), and that the two are sometimes conjoined, and thus give rise to mixed forms of the disease. The latter has always been a well-known disease in all countries; the first has been principally confined to India, and is that which is now desolating so large a portion of the globe.‡

* Epidemic Cholera, Spasmodic Cholera, Cholera Asphyxia Mordechi, of ot her authors.

† Bilious Cholera, and Cholera Morbus, of other authors.

‡ The disease by which the Israelites were so severely scourged in the time of David, as related by Josephus, bears a striking resemblance to the epidemic cholera, as will appear from the following passage:

"The prophet had no sooner received and reported David's answer, but the Israelites were seized with a most unaccountable distemper, that was still attended with certain death, and accompanied with accidents that baffled all the

Now it is worthy of remark, that the few descriptions of cholera which have been handed down to us from the Greek and Roman physicians, only correspond to the inflammatory or mixed forms, and no mention is made of the disease having ever occurred epidemically. Hippocrates and Galen must have been acquainted with the common cholera, since it is mentioned in different parts of their works; but they give no detailed account of it. The following description of Celsus is the most accurate which is found in the works of the ancients, and corresponds to some of the mixed cases which will be found in another part of this work.

"A visceribus ad intestina veniendum est quæ sunt et acutis et longis morbis obnoxia. Primoque facienda mentio est choleræ; quia commune id stomachi atque intestinorum vitium videri potest; nam simul et dejectio et vomitus est: præterque hæc inflatio est, intestina torquentur, bilis supra infraque erumpit, primum aquæ similis, deinde ut in ea recens caro lota esse videatur, interdum alba, nonnunquam nigra vel varia; ergo eo nomine morbum hunc χολεραν Græci nominarunt. Præter ea vero, quæ supra comprehensa sunt, sæpe etiam crura manusque contrahuntur, urget sitis, anima deficit: quibus concurrentibus, non mirum est si subito quis moritur; neque tamen ulli morbo minori momento succurritur."*

In the fifth chapter of the second book of Aretæus on the causes and symptoms of diseases, we find a tolerably accurate description of the inflammatory cholera; while the following, taken from the twentieth chapter of the third book of Cælius Aurelianus, rather corresponds to our mixed cases: he says,

"Precedit frequenta cholorícos stomachi gravedo atque tentio: anxietas; jactatio; vigiliæ; tormentum intestinorum cum sonitu, quem Græix borborismon vocant. Ventris dolor; atque per podicem venti fluor nihil relevans; suctationes fumosæ; nausea; salivarum fluor; gravedo thoracis cum membrorum de fectu; surgente passione jugis vomitus, et primo corrupti cibi; sicut frequenter occurrit, et humoris atque fellis flavii, dehinc vitellis ovorum similis; tunc prasii atque æruginosi; ultimo etiam nigri; ventris quoque turbatio cum dolore; et egestio vomitorum similis, hoc est spumora, et acerrima, cumfrequenti delectatione vomendi. Crescente passione aquasi atque tenuis liquoris sit egestio, et aliquando similis loturæ carnis. Feruntur etiam cum his humoribus plerumque subalbida desputa; sequitur etiam densitas pulsus, et articulorum frigus, atque vultus nigræ fuscatus; ardor atque sitis insatiabilis; spiratio celerrima; et contractio vel conductio membrorum, cum nervorumtensio, ac surarum et brachiorum. Præcordiorum etiam ad superiora raptus, cum dolore iliaco simili; aliquando etiam egestio ventris sanguinolenta, vultus in maciem atque tenuitatem deducti; oculi rubri; et in ultimo singultus."

Descriptions of the disease have also been given by numerous other medical writers both ancient and modern; among doctors, either to find a remedy or reason; but they died, in short, one upon the neck of another, and nobody knew how. *Some went off with gripes and torments, that despatched them in a trice; some with incurable faintnesses and langours, in despite of the physicians; others with vertigo, dimness of sight, suffocations, &c.; some, again, expired themselves in the very office of mourning for the death of others.* The mortality was so great, in fine, that, between break of day and dinner-time, there were swept away by this pestilence seventy thousand persons."—*The Works of Fl. Josephus, by* Sir Robert L'Estrange. London, 1702.

* Celsus de Medicina, lib. iv. chap. 2.

whom may be mentioned Paulus Agineta, Avicenna, Sydenham, Riverius, Hoffman, Cleghorn, Sauvages, and Girdlestone; none of whom, however, appear to have been acquainted with the pure catarrhal cholera.

The too famous epidemic known by the name of the black pest, which desolated Europe in the middle of the fourteenth century, has been supposed to resemble cholera.* It commenced in Upper India and the surrounding provinces, in the year 1346. It consisted of a vomiting of blood, attacked all conditions of people, and proved fatal very suddenly, or in two or three days. It spread from country to country, and within a year it included the whole of Asia. It then spread through Turkey into Greece, Sicily, Italy, and the opposite coast of Africa. In 1348 it had included the whole of Italy, except Milan and some parts of the Alps, and in the same year passed into Germany, Savoy, France, and Spain. In 1349, it had spread over the whole of the middle of Europe, except Brabant, which it affected but little, the north of Africa, England, Scotland, and Ireland; and in 1350 it extended over all the northern countries of the continent.† No accurate description of the symptoms of this epidemic have been preserved, so that we cannot say how far they agreed with those of cholera; but the mode of propagation appears to be similar in both, and it is remarkable that they spread over nearly the same tracts of country, and followed the same direction.

We are told by Sydenham that the cholera morbus prevailed with great severity in the year 1676. Among its symptoms are mentioned copious vomiting, spasms, small pulse, fainting, cold sweat; and the remedy he recommends is laudanum in strong cinnamon-water;‡ which shew that this was not the pure inflammatory cholera, but probably belonged to those mixed cases of the malady which I have described in the following pages, and which are of frequent occurrence in the epidemic visitations of India.

Sauvages defines the cholera to be "la complication de la diarrhee et du vomissement, ou de la diarrhee avec les nausees, ou du vomissement avec les tenesmes;" and he distinguishes no less than ten species, viz. cholera sec, chol. des pales cou-

* I understand that Heeran, the celebrated historian and professor at Gottingen, in a memoir lately read before the Royal Society of that city, but which I have not seen, has proposed the question, "Has the cholera which has reached Europe been known in this part of the world before?" and has given as his opinion, that the only disease which has borne any analogy to it is the pestilence mentioned above.

† See Historia di Matteo Villani, Cittadino Fiorentino, il quale continua l'Historia di Giovan Villani, suo fratello, &c. In Venetia, 1562. Lib. i. c. 1.

‡ Sydenham, Epistolæ de Morbis Epidemicis, &c. London, 1680.

leurs; cholera causé par des mauvais champignons; cholera causé par des poissons fossiles; cholera causé par impoison animal; cholera vermineux; cholera dissenterique; cholera gouteux; cholera crapuleux; cholera spontané, ou cholera morbus de Sydenham." It is impossible to say whether any of these correspond in their symptoms exactly to the catarrhal cholera; but none of them had ever occurred as a wide spreading epidemic.

In the Memoirs of the Italian Society of Sciences of Modena for 1805,* there is a description of cholera by Leopold M. Caldani, which more closely corresponds to the catarrhal form of the disease than any of those we have already mentioned. He says "frequent, unexpected, and impetuous vomiting, accompanied with copious dejections, whereby an individual, although very fat, often becomes lean visibly, so that, reduced as it were to a skeleton, he quickly dies, sometimes with severe abdominal pains, at other times with violent convulsions or seized by syncope, constitute that terrible disease which physicians call cholera morbus, or simply cholera." He points out the impropriety of limiting the term cholera to those cases in which the evacuations are bilious, and adds some interesting cases which appeared to have been produced by unwholesome milk, and in which the evacuations resembled that fluid.

Many of the cases of the very remarkable disease which raged in the Milbank Penitentiary in 1823, and which have been so ably described by Dr. Latham, exactly resemble the catarrhal cholera; nay, we cannot hesitate to consider them as cases of that disease produced by a local cause; and it is therefore necessary to bear in mind that, should the epidemic influence extend to this country, there is nothing in the climate hostile to its existence, or capable of arresting its progress.

The disease also which occurred among the boys of Mr. Day's school at Clapham, in August 1829,† strongly resembled the catarrhal cholera in its symptoms, but dissections proved it to be different; for, instead of the peculiar diseased appearances which are always presented by the gastro-enteric mucous membrane in cholera subjects, and which will be found described in the following sections, the mucous glands of these membranes in the fatal cases at Clapham were enlarged, giving an appearance of tuberculation or pustulation. This disease was caused by foul air from a drain which had been opened near the house, and was of a similar nature to the mor-

* Memorie di Matematica e di Fisica della Societa Italiana della Serenze, tomo 12, parte 2do, in 4to. Modena, 1805. Pp. 204, 224.

† See London Medical Gazette, August 1829.

bus mucosus, as described by Rœderer and Wagler,* which raged at Vienna sixty or seventy years ago. The only analogy which exists between these diseases and cholera is, that they have their seat in the same membrane; but the diseased action is evidently different, as shewn by the secretions which are the result of it; and the enlargement of the mucous glands, which appears to be the proximate cause of the morbus mucosus, has not been observed in cholera.

In New York, and indeed generally in the southern parts of the United States of North America, a disease exactly similar to the milder forms of the catarrhal cholera, is prevalent during the summer and autumn. It usually makes its attack at night, consists of vomiting and purging of thin watery fluids without bile, and produces sudden and great collapse, with cold skin. It has never occurred there as an epidemic, and is in general easily overcome (if combatted in time) by means of large doses of laudanum.†

I cannot finish this historical sketch without calling my reader's attention to the following remarkable points of analogy which are observable between different epidemic diseases. 1. Most of them are seated in the mucous membranes. An inflammatory catarrh of the air-passages, accompanied in some instances by looseness of the bowels, occurred as an epidemic throughout nearly the whole of Europe in 1580;‡ another of a similar nature spread over a great part of Europe in 1782;§ and the well-known influenza of 1803 consisted of a catarrh, with inflammation of the mucous membrane of the air-passages.|| 2. Epidemics have generally proceeded from the east to the west, which was the case with the peste noire, with the catarrh of 1782, with the influenza of 1803, which is said to have stretched from the confines of China to the shores of the Atlantic, and thence across to North America; and the same course is now pursued by the epidemic cholera. 3. All epidemics present the same capricious irregular characters in the mode of their propagation, sparing one spot to appear in a more distant one, following a zigzag course, suddenly stopping, and as suddenly and unexpectedly reappearing,

* Rœdereri et Wagleri, Tractatus de Morbo Mucoso.

† I am indebted for this information to Dr. Revere, whose distinguished talents and long experience in the United States give to his observations the highest value.

‡ Schneider de Catarrhis, lib. iv. cap. 5.

|| See Hamilton's Description of the Influenza in 1782.

§ Numerous other instances might be stated, did our limits permit: I need only refer to Sydenham's Epistolæ de Epidemicis, Parr's Medical Dictionary, Schneider de Catarrhis, Rœderer and Wagler.

proceeding sometimes slowly, sometimes rapidly, and so forth.*

The following conclusions appear to me naturally to arise out of the preceding details. 1st. That in almost all the European medical works, ancient as well as modern, we only find descriptions of the inflammatory and mixed forms of cholera; and that we must therefore conclude that these have, at all times, been the most prevalent kinds of the disease in this quarter of the globe. 2. That the catarrhal cholera (viz. that which is now gradually approaching from India,) has never before made its appearance in the form of an epidemic in Europe; but that sporadical cases have been occasionally observed and described in different European countries, and also in the United States of North America. 3. That epidemic cholera is not contagious,† but exactly resembles other epidemics in the mode of its propagation, and in its general features.

(To be continued.) p. 456.

---

### CONNEXION BETWEEN THE PLACENTA AND UTERUS.

* "Adeolento tracto morbi epidemici integras interdum regiones pervagantur, ut presso quasi pede eorum vestigia legere et consectari nobis liceat. Neque vero ventorum iter aut linearum quamdam rectam sed alios tramites, nondum satis extricabiles, lineamque obliquam sequuntur, factis interdum in progressu saltibus."—Rœdereri et Wagleri, *de Morbo Usu*, sect. 1.

† This important question shall be fully discussed in a future section.

